# TRPV1 and the MCP-1/CCR2 Axis Modulate Post-UTI Chronic Pain

**DOI:** 10.1038/s41598-018-24056-0

**Published:** 2018-05-08

**Authors:** John M. Rosen, Ryan E. Yaggie, Patrick J. Woida, Richard. J. Miller, Anthony J. Schaeffer, David J. Klumpp

**Affiliations:** 1 Departments of Urology, Feinberg School of Medicine, Northwestern University, Chicago, Illinois 60611 USA; 2 Microbiology-Immunology, Feinberg School of Medicine, Northwestern University, Chicago, Illinois 60611 USA; 3 Department of Pharmacology, Feinberg School of Medicine, Northwestern University, Chicago, Illinois 60611 USA; 4 Present Address: Division of Pediatric Gastroenterology, Children’s Mercy Hospital, Kansas City, MO USA

## Abstract

The etiology of chronic pelvic pain syndromes remains unknown. In a murine urinary tract infection (UTI) model, lipopolysaccharide of uropathogenic *E. coli* and its receptor TLR4 are required for post-UTI chronic pain development. However, downstream mechanisms of post-UTI chronic pelvic pain remain unclear. Because the TRPV1 and MCP-1/CCR2 pathways are implicated in chronic neuropathic pain, we explored their role in post-UTI chronic pain. Mice were infected with the *E. coli* strain SΦ874, known to produce chronic allodynia, and treated with the TRPV1 antagonist capsazepine. Mice treated with capsazepine at the time of SΦ874 infection failed to develop chronic allodynia, whereas capsazepine treatment of mice at two weeks following SΦ874 infection did not reduce chronic allodynia. TRPV1-deficient mice did not develop chronic allodynia either. Similar results were found using novelty-suppressed feeding (NSF) to assess depressive behavior associated with neuropathic pain. Imaging of reporter mice also revealed induction of MCP-1 and CCR2 expression in sacral dorsal root ganglia following SΦ874 infection. Treatment with a CCR2 receptor antagonist at two weeks post-infection reduced chronic allodynia. Taken together, these results suggest that TRPV1 has a role in the establishment of post-UTI chronic pain, and CCR2 has a role in maintenance of post-UTI chronic pain.

## Introduction

Urinary tract infections (UTI) are the second most common bacterial infection, with about 50% of women developing an infection during their lifetime and about 13% of women experiencing recurrent infections^1^. These acute pain events may be a precursor to chronic disease as a history of UTI is associated with interstitial cystitis /bladder pain syndrome (IC) characterized by chronic pelvic pain^2^. Like other chronic pain conditions, IC is associated with psychological comorbidities, including depression and reduced quality of life^3^.

Uropathogenic *E. coli* (UPEC) are the most common bacteria causing UTI. Once infection is established, the host mounts an immune response to clear the bladder of the invading pathogen^4^. This is achieved through toll-like receptor 4 (TLR4) recognition of bacterial lipopolysaccharide (LPS) leading to expression of IL-6 and IL-8 and recruitment of neutrophils to infected tissue^4^. A model of murine UTI demonstrated that pain is abolished in TLR4 deficient mice and that the O-antigen of LPS modulates infection-induced pain^5,6^. UPEC strain NU14 harboring a deletion of the wz* gene cluster that encodes O-antigen exhibited a chronic pain phenotype by inducing chronic pelvic allodynia that persisted after bacterial clearance, whereas complementation of the UPEC wz* deletion mutant with various wz* gene clusters modulated the bacterial pain phenotype^6^. This pain modulation included restoring the acute pain phenotype of wild type NU14 by complementing NU14Δwz* with an NU14wz* fosmid or suppressing pain phenotype of NU14Δwz* with a wz* fosmid derived from asymptomatic bacteriuria-associated *E. coli* strain 83972, a strain that exhibits an analgesic phenotype^7^. However, the host mechanisms that allow for the establishment and maintenance of post-UTI chronic pain are still unknown.

Transient receptor potential vanilloid type-1 (TRPV1) is an ion channel expressed on bladder afferent sensory neurons and urothelium. Activation of TRPV1 through heat, protons or molecular ligands such as capsaicin, allows for the transport of cations into the cell and is associated with neuropathic pain^8,9^. Absence of TRPV1 inhibited pelvic pain in chemically induced cystitis^5^, but the role of TRPV1 in UTI-associated pain has not been described.

The chemotactic cytokine receptor 2 (CCR2) is a G-protein coupled receptor that when engaged by its ligand, monocyte chemoattractant protein-1 (MCP-1) or CCL2, has a role in monocyte recruitment in response to inflammation^10^. MCP-1 expression and CCR2 binding are up-regulated in response to compression of the dorsal root ganglia (DRG), a model for neuropathic pain^11^. When treated with Freund’s adjuvant (CFA), CCR2-deficient mice had reduced mechanical allodynia compared to wild type controls^10^. These results suggest that the MCP-1/CCR2 axis has a role in neuropathic pain induction, yet its role in post-UTI pelvic pain response has yet to be explored.

We examined both pelvic pain and cognitive behaviors in mice following experimental acute UTI with *E. coli* strain SΦ874 and characterized the involvement of TRPV1 and CCR2. Our results show that when infected mice are treated with a TRPV1 antagonist at the initial time of infection, they fail to develop chronic pelvic pain or behavioral changes associated with anxiety and depression. We also observed a reduction in pelvic pain when mice infected with *E. coli* SΦ874 or the clinical *E. coli* strain NU23 were treated with a CCR2 antagonist two weeks post-infection. Our results suggest that in post-UTI chronic pain, TRPV1 has a role in the establishment of chronic pelvic pain while MCP-1/CCR2 has a role in the maintenance of pain.

## Results

### TRPV1 is involved in the establishment of post-UTI chronic pain

**Figure 1:**
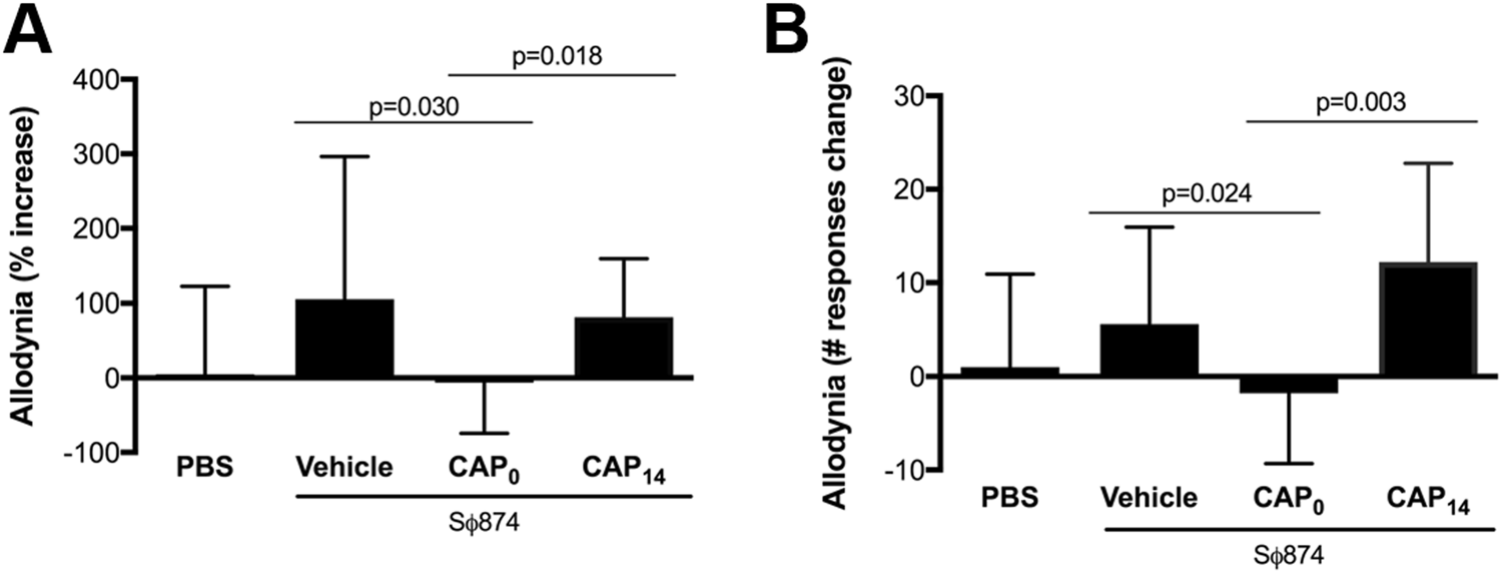
TRPV1 mediates establishment of pelvic allodynia in post-UTI chronic pain. UTIs were induced in female mice by transurethral instillation of *E. coli* strain SΦ874 or saline (PBS, n = 13). SΦ874 infected mice (n = 13) were administered 30 mg/kg capsazepine i.p. at at PID 0 (CAP_0_, n = 13), at PID 14 (CAP_14_, n = 5), or treated with vehicle at PID 0 (n = 13). Mice were then evaluated for allodynia by application of von Frey filaments to the pelvic area at PID 0 and PID 15. Values represent mean ± S.D. (**A**) Reduced allodynia of SΦ874-infected mice administered capsazapine at PID 0 but not in mice receiving capsazapine at PID 14. The percent change of behavioral responses relative to baseline at PID 0. (**B**) Numerical change in behavioral responses relative to baseline (*P < 0.05).

To explore the role of TRPV1 in post-UTI chronic pain, we examined the effects of the TRPV1 antagonist capsazepine in mice infected with the *E. coli* strain SΦ874. SΦ874 lacks LPS O-antigen and was previously reported to induce chronic murine mechanical allodynia following UTI that persists for more than 35 days^6^. Because SΦ874-induced chronic allodynia is established by day 5 and persists for weeks, assessments in this study were conducted 14 days post UTI to evaluate the chronic pain state. Female C57BL6/J mice (wild type, WT) were instilled via transurethral catheter with either SΦ874 or saline. Mice were also treated with intraperitoneal capsazepine or vehicle at the time of infection or two weeks after the initial infection (post-infection day 14, PID 14). Mechanical allodynia was then measured at PID 15 in response to von Frey filaments applied to the pelvic region^6^. Mice treated with capsazepine at the time of SΦ874 infection showed significantly reduced allodynia relative to mice treated with capsazepine at PID14, both in terms of change from baseline and individual responses (Fig. 1A and B, respectively). These results show that post-UTI pain was altered by targeting TRPV1 only when capsazepine was given at the time of infection, suggesting that TRPV1 plays a role in the establishment, of post-UTI chronic pain but not in maintenance of post-UTI pain.

**Figure 2:**
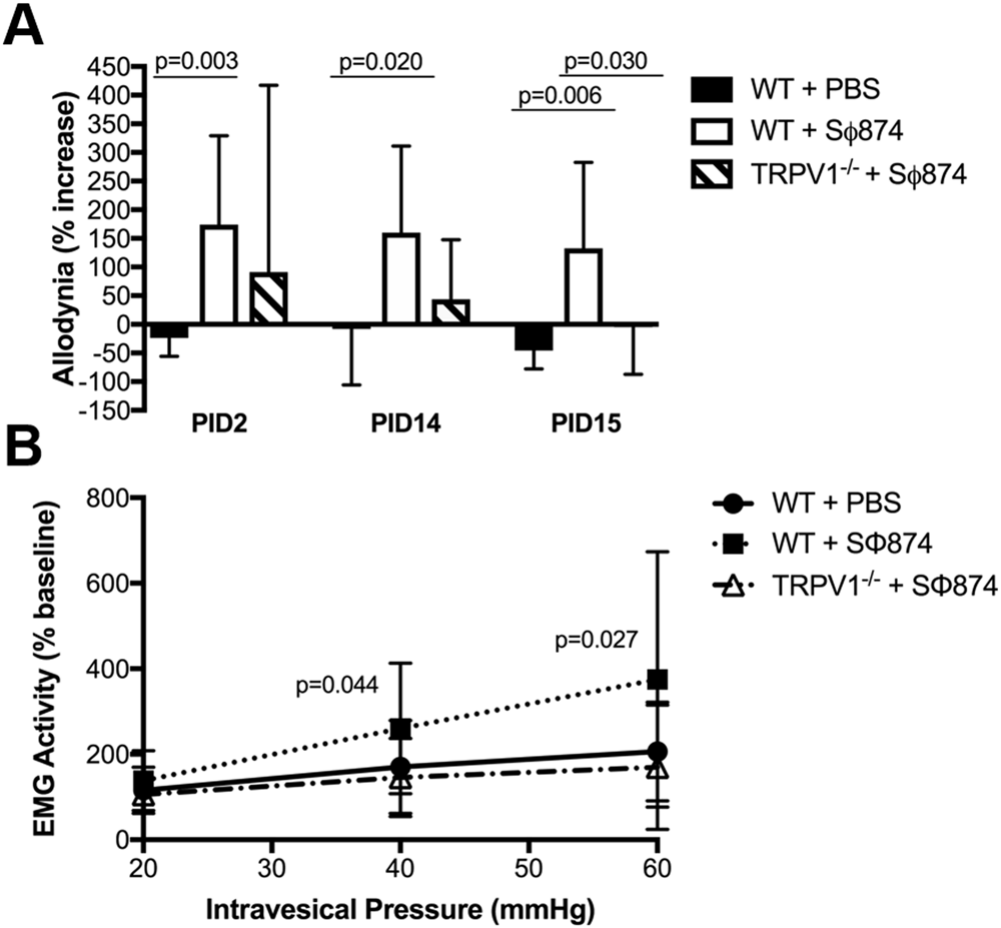
TRPV1-deficient mice do not develop post-UTI chronic pain. Values represent mean ± S.D. (**A**) Female WT or TRPV1^−/−^ mice were instilled with saline or *E. coli* strain SΦ874, and mechanical allodynia was quantified on PID 2, PID 14, and PID 15. SΦ874 induced significant allodynia in WT mice (n = 8, P < 0.05) relative to saline-treated mice (n = 8), whereas TRPV1^−/−^ SΦ874-treated mice did not (n = 7). (**B**) VMR in response to bladder distension with saline at PID 15 after instillation of SΦ874 or PBS. Relative to saline-treated wild type mice, SΦ874 induced increased EMG activity in wild type mice that was not induced in TRPV1^−/−^ mice (n = 5, *P < 0.05).

To confirm the role of TRPV1 in post-UTI chronic pelvic pain, we examined the development of chronic pain in TRPV1-deficient mice. Wild type mice initially infected with SΦ874 displayed significantly increased mechanical allodynia at PID 2, 14, and 15, while SΦ874-infected TRPV1-deficient mice did not (Fig. 2A). To confirm the behavioral measure of referred pelvic pain using allodynia, we also examined the role of TRPV1 in pelvic pain by bladder visceromotor response (VMR). VMR measures the electromyographic motor reflex response to noxious bladder distention^12^. At PID 15, wild type mice infected with SΦ874 had a significantly higher VMR response than saline-treated wild type mice at pressures of 40 and 60 mmHg (Fig. 2B), indicating persistent visceral hypersensitivity in post-UTI pelvic pain induced by SΦ874. In contrast to wild type mice however, TRPV1-deficient mice did not exhibit visceral hypersensitivity following infection with SΦ874. Thus, consistent with the findings using capsazepine to target TRPV1, TRPV1-deficient mice support a role for TRPV1 in the establishment of post-UTI chronic pain induced by SΦ874.

### TRPV1 mediates post-UTI depression

**Figure 3:**
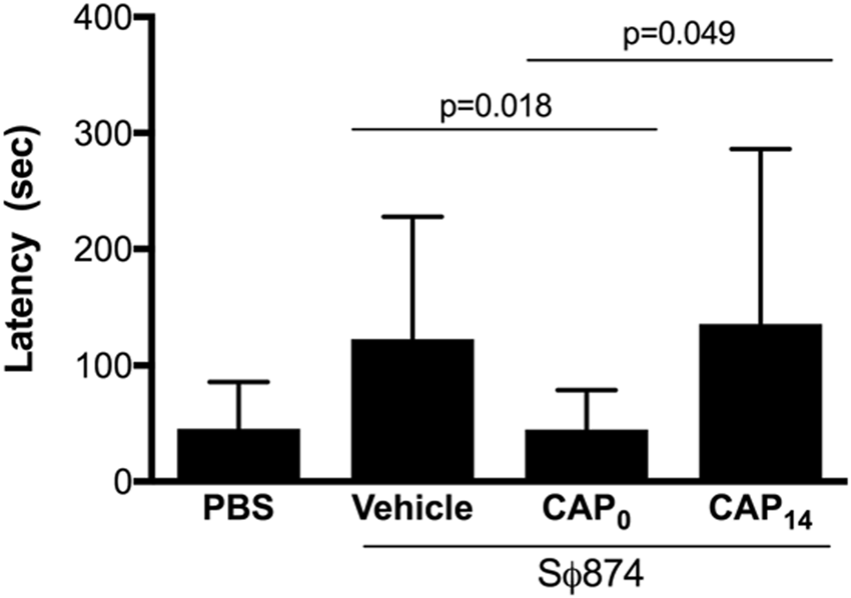
TRPV1 mediates depressive behavior associated with post-UTI chronic pain. UTIs were induced in female mice by transurethral instillation of *E. coli* strain SΦ874 or saline (PBS, n = 13). SΦ874 infected mice (n = 13) were administered 30 mg/kg capsazepine i.p. at PID 0 (CAP_0_, n = 13), at PID 14 (CAP_14_, n = 5), or treated with vehicle at PID 0 (n = 13). Mice were then assessed for depressive behavior by quantifying feeding latency in novelty-supressed feeding assay at PID 15. SΦ874-infected mice receiving vehicle exhibited significantly increased latency (P < 0.05) relative to saline-treated mice that was prevented in mice receiving capsazepine administered at PID 0 but not altered by capsazepine administered at PID 14. Values represent mean ± S.D.

Patients suffering from IC also typically suffer co-morbid anxiety and depression that are a manifestations of chronic pain^13^. Therefore, we examined if post-UTI chronic pain is also associated with cognitive dysfunction in mice consistent with depression and whether TRPV1 is a potential mediator of any dysfunction. As a measure of depressive/anxious behaviors in mice, we employed novelty suppressed feeding (NSF), an assay where depressive behavior is detected as increased latency of a hungry mouse to approach food when placed into a novel environment^14^. SΦ874-infected wild type mice were treated with capsazepine or vehicle via intraperitoneal injection either at the time of infection or treated with capsazepine at PID 14 and assessed by NSF. SΦ874-infected mice showed a significant increase in latency relative to saline-treated mice, suggesting that SΦ874-induced chronic pain is also associated with depressive/anxious behavior (Fig. 3). In contrast, mice treated with capsazepine at PID 0 did not develop increased latency. However, SΦ874-infected mice treated with capsazepine at PID 14 showed increased latency times similar to vehicle-treated, SΦ874-infected mice. Like the development of post-UTI pain itself, these data suggest that TRPV1 mediates the establishment of cognitive dysfunction consistent with depression that associated with post-UTI chronic pain, but TRPV1 is dispensable for maintenance of depressive behaviors.

### MCP-1/CCR2 expression is modulated by LPS O-Antigen

**Figure 4:**
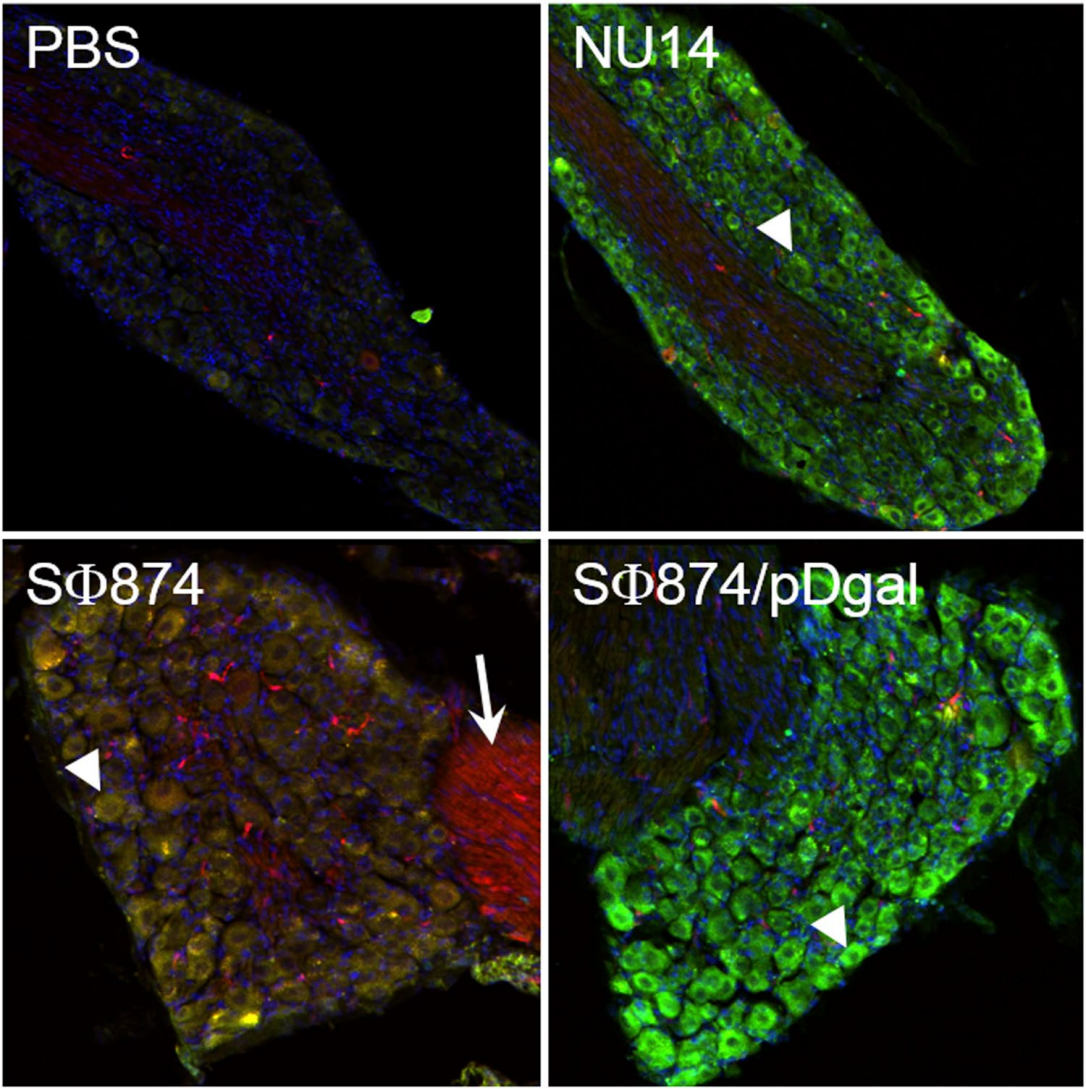
Post-UTI chronic pain is associated with MCP1 induction. MCP-RFP/CCR2-GFP mice were instilled with PBS or infected with *E. coli* strain NU14, SΦ874 or SΦ874/pDgal. Isolated sacral DRGs of saline-instilled mice have low RFP and GFP expression. GFP expression was marked in DRG cell bodies of mice infected with either NU14 or SΦ874/pDgal (arrowheads). DRGs of SΦ874-infected mice expressed RFP in fibers (arrow) colocalized with neuronal cell bodies expressing GFP (yellow, arrowhead).

The chemokine MCP-1 and its receptor CCR2 have been implicated in neuropathic pain^10,15,16^. Therefore, we evaluated the potential role of MCP-1/CCR2 post-UTI chronic pain by evaluating expression in the dorsal root ganglia (DRG) of bitransgenic reporter engineered to express MCP-1 and CCR2 as fusion proteins with red fluorescent protein (RFP) and green fluorescent protein (GFP), respectively^17^. Reporter mice were instilled with saline, with UPEC isolate NU14 that causes acute pelvic allodynia in mice, with SΦ874, or with SΦ874/pDgal, bearing a plasmid encoding *Klebsiella* O-antigen that suppresses the pain phenotype of SΦ874^6,18^. DRGs were isolated from reporter mice at times corresponding to allodynia (i.e., PID 2 for saline controls and NU14-infected mice, PID 14 for SΦ874- and SΦ874/pDgal-infected mice) and then examined for MCP-1-RFP and CCR2-GFP expression by confocal fluorescence microscopy in frozen sections (Fig. 4). Saline-treated control mice showed no expression of GFP or RFP in sacral DRGs. SΦ874 induced expression of both RFP and GFP, consistent with expression of MCP-1 and CCR2. CCR2-GFP was detected in DRG cell bodies, and MCP-1-RFP was found to co-localize with CCR2-GFP in DRG cell bodies as well as along DRG axons. In contrast, DRGs of mice infected with either NU14 or SΦ874/pDgal expressed only low levels of MCP-1-RFP but high levels of CCR2-GFP in DRG cell bodies. These results suggest that both MCP-1 and CCR2 are upregulated in response to *E. coli* that induce post-UTI chronic pain, but bladder infection by *E. coli* generally induces CCR2 expression, regardless of *E. coli* pain phenotype.

### CCR2 promotes the maintenance of post-UTI chronic pain

**Figure 5:**
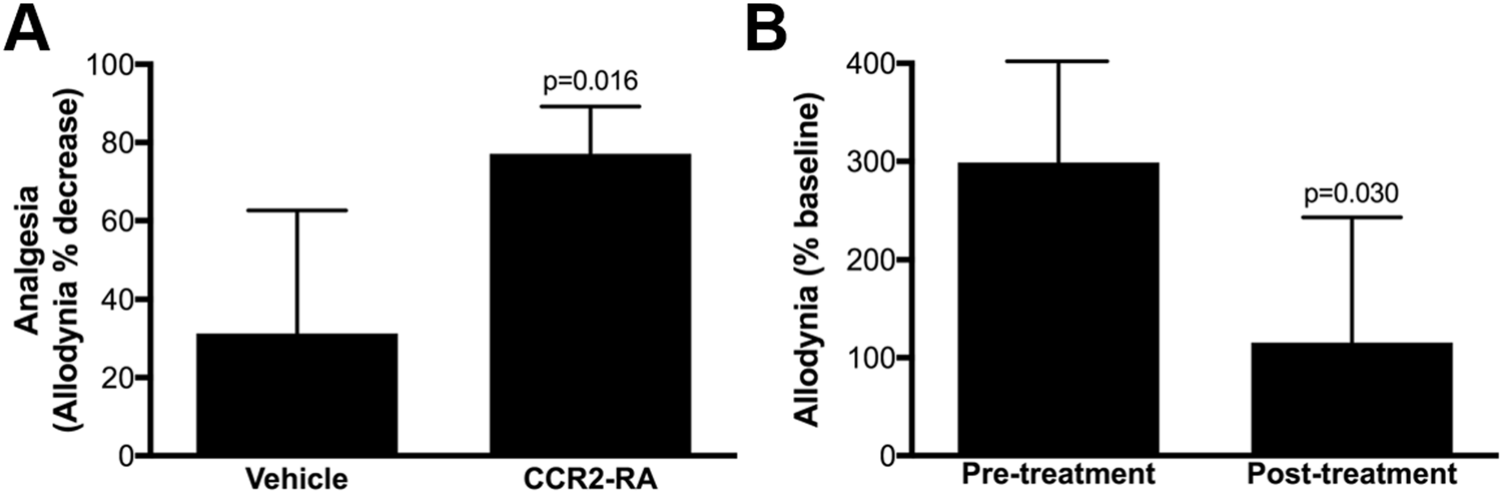
CCR2 mediates post-UTI chronic pain. Values represent mean ± S.D. (**A**) Female B6 mice were infected with *E. coli* strain SΦ874. On PID 16, mice received i.p. injection of 10 mg/kg CCR2-RA or vehicle (n = 5 each group). Mice receiving CCR2-RA exhibited significantly decreased allodynia (*P < 0.05). (**B**) Mice were infected with UPEC strain NU23 (n = 8). On PID 14, female B6 mice were evaluated for allodynia relative to baseline. Mice were then re-evaluated after administration of 20 mg/kg CCR2-RA. CCR2-RA significantly reduced allodynia induced by NU23 (*P < 0.03).

Since MCP-1 expression was upregulated in response to SΦ874 infection, we hypothesized that MCP-1 plays a functional role in post-UTI chronic pain. To test this hypothesis, SΦ874-infected mice were treated on PID 16 with either vehicle or the CCR2 antagonist (R)-4-Acetyl-1-(4-chloro-2-fluorophenyl)-5-cyclohexyl-3-hydroxy-1,5-dihydro-2H-pyrrol-2-one (CCR2-RA), previously shown to reduce neuropathic pain^19^. SΦ874-infected mice achieved significantly higher analgesia following CCR2-RA treatment, relative to vehicle-treated mice (Fig. 5A). To corroborate these findings, wild type mice were infected with NU23, a clinical UPEC isolate from the urine of a cystitis patient^20^. On PID14, pelvic allodynia was measured prior to and then 60 minutes following treatment with CCR2-RA. NU23-infected mice showed decreased allodynia after treatment with CCR2-RA, relative to the same mice prior to treatment (Fig. 5B), suggesting that CCR2 plays a role in chronic post-UTI pain induced by NU23. Moreover, these data suggest that the MCP-1/CCR2 axis is required for maintenance of post-UTI chronic pain.

## Discussion

Previous studies showed that UPEC induce pelvic allodynia consistent with bladder-associated pelvic pain, and this allodynia is dependent upon TLR4 and modulated by LPS O-antigen independent of bladder inflammation^6,18^. Despite this understanding of distal factors triggering painful bladder infection, the downstream molecular mediators of UTI pain are still not well understood. TRPV1 is a factor mediating spinal c-fos expression and reflex bladder contractions in a model of acute cystitis^21^. TRPV1 and CCR2 have both been shown to be activated in models of neuropathic pain^15,16^, however their role in the development of post-UTI chronic pelvic pain initiated by infection has not been explored previously. Here we used both pharmacologic and genetic approaches to demonstrate the requirement for TRPV1 in post-UTI chronic pelvic pain initiated by infection with the K12 strain SΦ874 (Figs 1 and 2), a strain shown to cause chronic allodynia that persists for weeks and months (ref.^6^ and Rudick and Klumpp, unpublished observations). Indeed, TRPV1 was required for both mechanical allodynia and for visceral hypersensitivity manifested as increased VMR in response to bladder distension (Figs 1 and 2) as well as increased NSF latency consistent with depressive behavior (Fig. 3). However, TRPV1 may play a temporally-specific role in the development of post-UTI chronic pelvic pain and depression because the TRPV1 antagonist capsazepine reduced allodynia and NSF latency only when administered at the onset of infection (Figs 1 and 3). Subsequent administration of capsazepine had no effect on established pelvic pain or depressive behavior. Together these data suggest that TRPV1 mediates establishment of *E. coli*-induced post-UTI chronic but does not play a role in maintenance of chronic pelvic pain.

CCR2 appears to play a temporally distinct role in the development of post-UTI chronic pain from that of TRPV1. CCR2 is induced in sacral DRGs during post-UTI chronic pain (Fig. 4). However, in contrast to TRPV1, administration of a CCR2 antagonist in mice with post-UTI chronic pain provided analgesia in mice with allodynia induced by either a K12 strain or a UPEC isolate (Fig. 5). These findings demonstrate that CCR2 mediates maintenance of post-UTI chronic pain but also identify key differences with other urologic pain models. For example, we previously observed that TLR4-dependent acute UTI pain did not correlate with bladder inflammation^18^. Similarly, *E. coli* strains with distinct bacterial pain phenotypes, ranging from chronic pain to analgesia, did not elicit differential bladder inflammation or pathology^6^. These findings are somewhat in contrast to an *E. coli*-induced model of chronic prostatitis/chronic pelvic pain syndrome (CP/CPPS) that is mediated by a Th1/Th17 immune mechanism^22^. Conversely, SΦ874 induced both CCR2 and its cognate ligand, MCP-1, within DRGs (Fig. 4), and TRPV1 is expressed on bladder afferent C fibers^23^. Thus, while we cannot exclude the possibility of additional cellular influences, the results reported here suggest that post-UTI chronic pain is initially mediated primarily by mechanisms intrinsic to the bladder sensory system.

MCP-1/CCR2 axis induction within sacral DRGs has implications for bacterial pain phenotypes and clinical responses to bladder infection. SΦ874 has a chronic pain phenotype and induced both MCP-1 and CCR2, whereas the acute pain phenotype of NU14 was associated only with CCR2 induction (Fig. 4). Together these observations suggest that MCP-1 induction during bladder infection is a key event in the acute-to-chronic pain transition and a defining feature underlying the chronic pain phenotype of *E. coli*. Future studies will define *E. coli* factors that mediate general CCR2 induction and differential MCP-1 induction and thereby identify virulence determinants of the acute-to-chronic pain transition. However, in the meantime CCR2 induction by *E. coli* may also have clinical implications. We note that NU14 and SΦ874/pDgal both induce DRG CCR2 despite exhibiting distinct pain phenotypes of acute and null pain, respectively. Although SΦ874/pDgal is cleared rapidly from the bladder, it nonetheless induces sustained CCR2 expression. This raises the possibility that a subset of patients with acute UTI or asymptomatic bacteriuria may nonetheless become sensitized by CCR2 induction along the bladder sensory pathway. A subsequent infection or other insult sufficient to trigger MCP-1 could then lead to chronic pelvic pain. There is clinical support for such a possibility because IC patients often have a history of UTI preceding development of chronic pelvic pain^24^. In addition, elevated MCP-1 levels in prostatic fluid have been associated with CP/CPPS^25^. Thus, events that induce the MCP-1/CCR2 axis may trigger pelvic sensory hyper-excitability in general, suggesting CCR2 as a general therapeutic target for urologic chronic pelvic pain syndromes.

## Materials and Methods

### Ethics statement

Mice were housed in Northwestern’s Center for Comparative Medicine (CCM) and were cared for only by trained facility personnel. Mice were housed with environmental enrichment and monitored frequently and routinely to confirm good health and absence of visible stress. All research protocols were approved by the Northwestern IACUC and followed NIH guidelines to minimize pain or stress, and all laboratory personnel were appropriately trained and certified, including euthanasia training. All experiments were performed in accordance with relevant guidelines and regulations. All experiments were designed to obtain statistically significant findings with the minimum number of mice, and experiments were terminated as soon as possible. In prior studies of UTI-induced pelvic allodynia, statistical significance was achieved in n = 5–10 mice^18,6^.

### Bacterial strains and culture

NU23 is a clinical isolate of *E. coli* obtained from a patient with acute UTI^20^. SΦ874 is a K-12 isolate and in some experiments carried the plasmid pDGal (a.k.a. pWQ288) encoding *Klebsiella pneumoniae* O-antigen serotype 02a^6^. *E. coli* strains were cultured in Luria broth at 37°; NU23 was cultured under static conditions, whereas SΦ874 was cultured with shaking.

### Experimental UTI

Female C57BL/6 J mice and TRPV1-deficient mice (8–12 weeks) were purchased from The Jackson Laboratory. MCP-1/CCR2 reporter mice (MCP1::MCP1-mRFP1;CCR2::CCR2-EGF^17^) were bred in CCM facilities. Mice were infected with *E. coli* via transurethral catheter by instilling 10 µl containing 10^8^ CFU in a non-reflux UTI model^26^.

### Confocal microscopy

Reporter mice were perfused and fixed with 4% paraformaldehyde. Sacral dorsal root DRGs (S1–S3) were dissected, cryoprotected through a sucrose series (10–30%) and frozen in OCT. DRG levels were selected based on a prior study demonstrating altered responses from S1–S3 in mice with chronic pain post-SΦ874 infection^6^. Frozen sections were hydrated, cover slipped, and visualized by confocal microscopy.

### Allodynia

Allodynia was quantified in response to von Frey filament stimulation to the pelvic region or the hind paw by a blinded tester^18^. Briefly, mice were adapted to the test chamber environment (5–10 min.) and von Frey filaments were applied 10 times each to the pelvic region, moving the stimulus with each successive fiber application to avoid wind up.

### Visceromotor response (VMR)

VMR was used to quantify distension-evoked bladder nociception in response to bladder filling as a measure of visceral pain. Briefly, myoelectric activity was recorded in response to bladder distension from electrodes implanted in the external oblique muscle as described previously^27,28^. Mice were maintained under light anesthesia with isoflurane, and muscle activity was recorded in response to filling with saline at intravesical pressures of 20–60 mm Hg. Data were captured and quantified in Spike2 software (Cambridge Electronic Design).

### Novelty-suppressed feeding assay

The novelty-suppressed feeding assay was used to assess depressive-like behaviors in mice^29^. Briefly, mice were deprived of food 24 hours before the testing period and deprived of water 2 hours before the testing period. For testing, a mouse was placed into the corner of a “novel environment,” a large chamber of 51 × 51 × 17 cm with a mouse chow pellet in the middle. Latency was measured as the interval between placing a mouse in the chamber and the time to approach the food pellet.

### Statistical approaches

Results were expressed as means ± SD. When the data is compared between two groups, they were analyzed with the student t-test; while data compared from more than two groups, were analyzed by one-way ANOVA followed by a post-test comparison using either Bonferroni’s or Tukey’s multiple comparison test. All analysis was done using Prism software (GraphPad). A value of P < 0.05 was considered statistically significant.

### Data availability

All data are available upon requested.
